# Supplementary material for: The mechanism of tidal triggering of earthquakes at mid-ocean ridges
Source: Nat Commun. 2019 Jun 7;10:2526. doi: 10.1038/s41467-019-10605-2 (PMC6555822; doi:10.1038/s41467-019-10605-2)
Supplement: Supplementary file 1 — Supplementary Information [file 41467_2019_10605_MOESM1_ESM.pdf]

Supplementary Information

The Mechanism of Tidal Triggering of Earthquakes at Mid-Ocean Ridges

Scholz *et al.*

Supplementary Figures

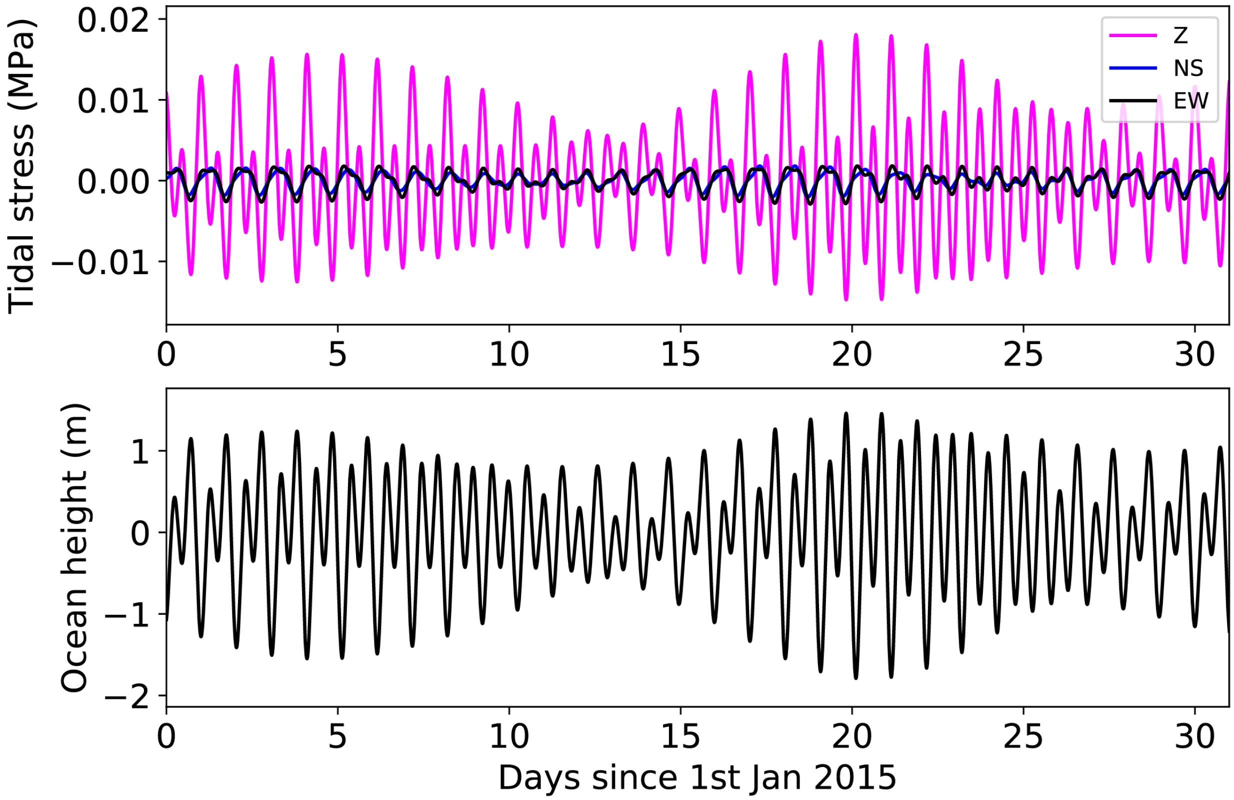

Supplementary Figure 1. Tidal stresses and ocean height calculated for Axial Volcano for January 2015.

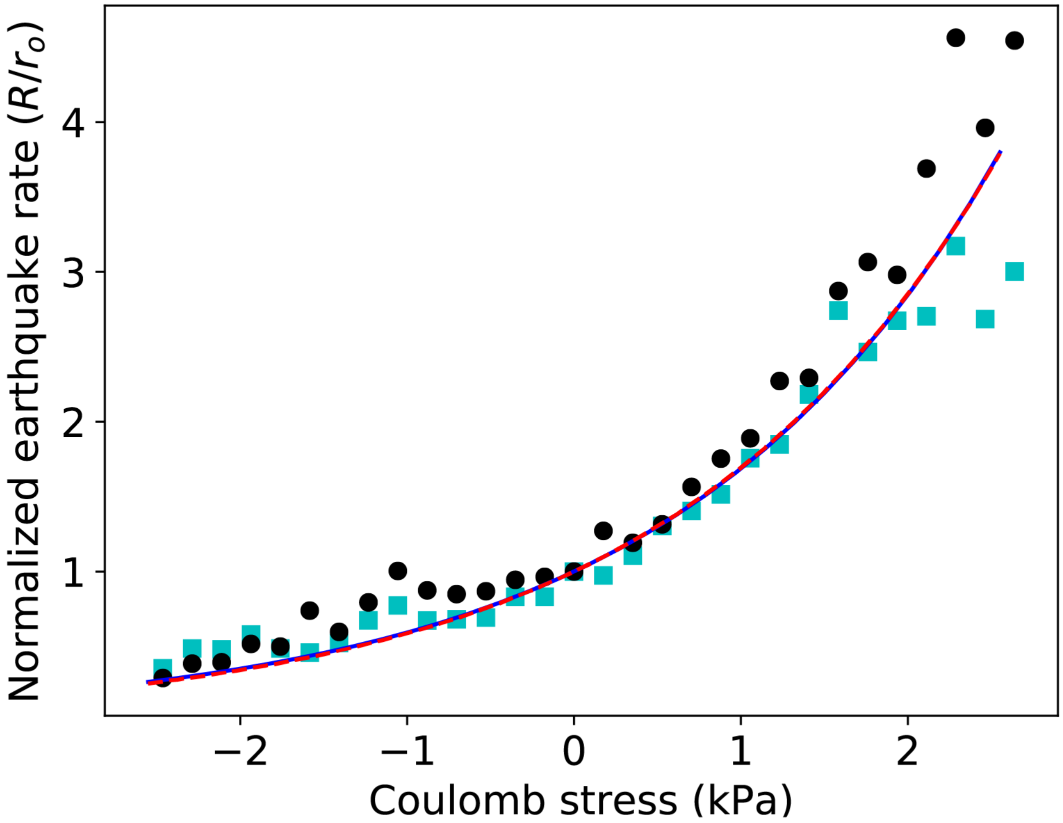

Supplementary Figure 2. Hysteresis test of the seismicity data. Cyan square is rising tide while black circle is falling tide. Curves are the same as in Fig. 5. Slight discrepancy at higher end probably reflects poor sampling at high stresses (see Supplementary Fig. 3).

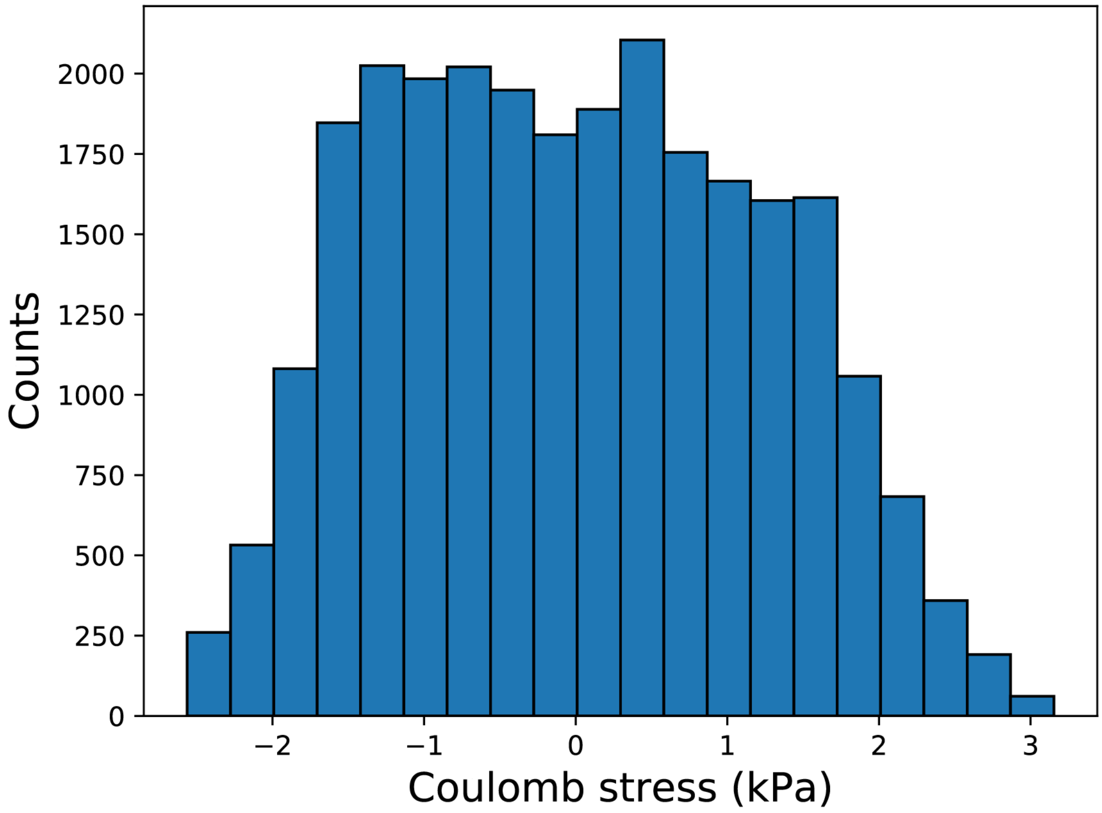

Supplementary Figure 3. Histogram of the tidal Coulomb stress amplitudes.

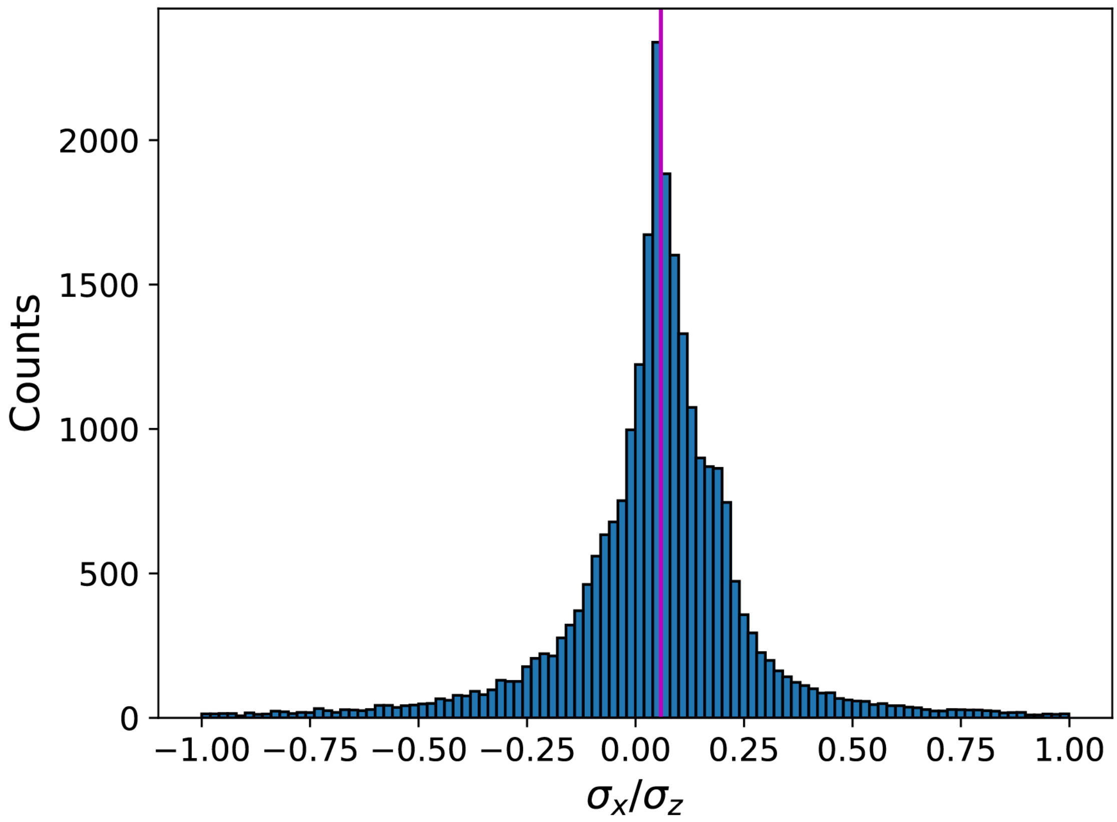

Supplementary Figure 4. Histogram of the ratio between horizontal and vertical tidal stresses. Horizontal stress is taken as the average of the north-south and east-west tidal stresses (Supplementary Fig. 1). Magenta line shows the median value of 0.058.

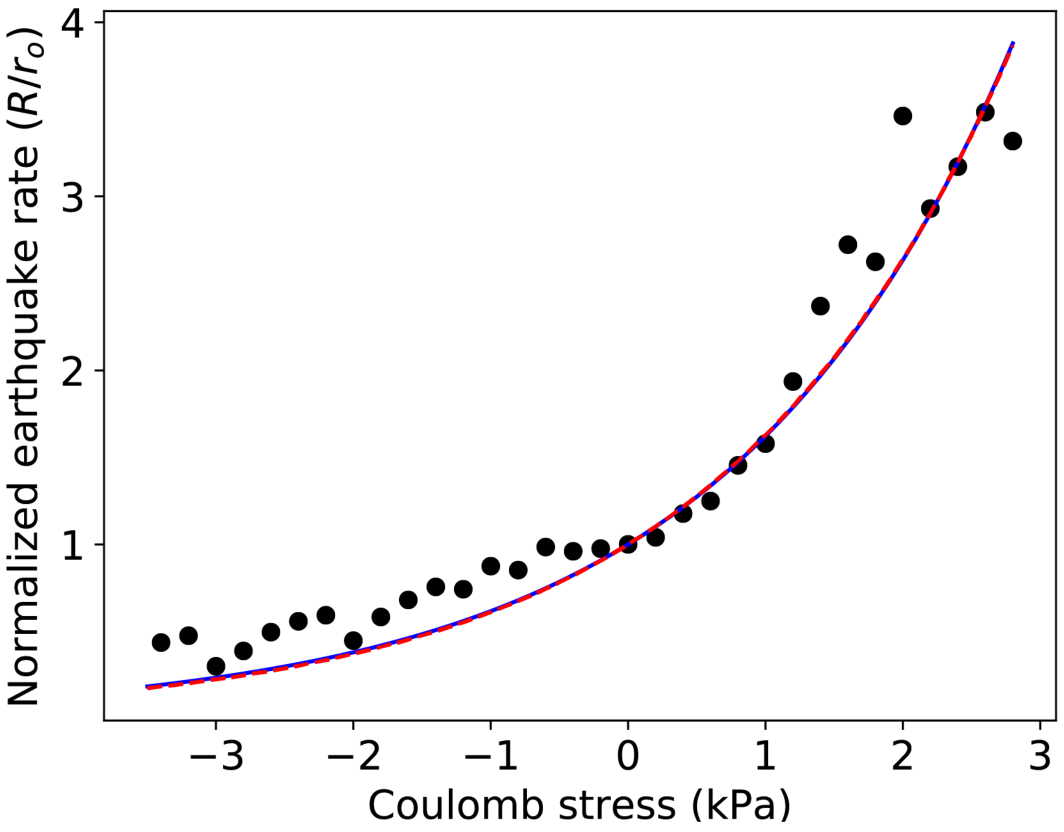

.

Supplementary Figure 5. Normalized seismicity rate change vs. change in Coulomb stress. The combined Coulomb stress contribution from the tidal vertical and horizontal stresses is calculated using $\chi_1 = 0.133$, which relates the tidal vertical stress to the Coulomb stress change on the fault and $\chi_2 = 0.740$, which relates the tidal horizontal stress to the Coulomb stress change on the fault (see Methods). Horizontal stress is taken as the average of the north-south and east-west tidal stresses (see Supplementary Fig. 1). Lines show fit using the rate-state friction model (blue) and the stress corrosion model (red). For the rate-state model, using 7.2 MPa for σ yields A=0.0003. For the stress corrosion model, the best fitting stress drop is $0.04<\Delta\tau<0.09$ MPa for $22<n<44$[40].
